# Fifteen Uses for the Indiarubber Condom

**Published:** 1875-07

**Authors:** H. Otis Hyatt

**Affiliations:** Kinston, N. C.


Fifteen Uses for the Indiarubber Condom.—*By H. Otis Hyatt, Kinston, N. C.—Read before the Eastern (N. C.) Medical Association.*—Permit me to call your attention to fifteen uses for the condom, or so-called male safe. The condom, as you are aware, was originally designed as a means of preventing conception, and as a protection against venereal diseases. The use of it for anti-conception purposes has no doubt caused a great deal of suffering among women, producing cervicitis and a long train of nervous ills. It acts as a dark, impenetrable veil, that shields the infamy of those whom we often believe pure, and as a source of temptation, to those who are lascivious, to sin without fear of detection. Its evils are great, but every cloud has its silver lining, and every evil its good; so I hope to show you that even the condom may often be turned to good account.

First. As a means of applying heat or cold to inflamed surfaces, filled with ice, and the end tied, it will be found a most useful application to an inflamed eye, or the forehead of a fever patient.

Second. To arrest bleeding from the nose, which it will do as effectually as plugging by Bellocq's canula or by the insufflation of the persulphate of iron. To do this a small size condom is tied over the end of a Davidson's syringe nozzle near its point, the pendant or baggy portion is pushed into the nose, as far back as the posterior nares; it is then filled with water which causes it to expand in all possible directions, and to fill up the entire nasal cavity, producing an arrest of hæmorrhage by direct pressure upon the bleeding surfaces. A cord is now securely tied around the condom just anterior to the syringe nozzle from which it is liberated. It can remain in the nose as long as is desirable.

Third. Three large condoms filled with ice, and placed one above the other, will answer the purpose of a spinal ice bag, or filled with hot water and placed up and down the spine, a most useful and reliable means of arresting uterine hemorrhage. I have frequently arrested hemorrhage from the non-pregnant uterus by the application of heat to the spine, after the failure of large doses of ergot, and of quinine. I may here remark that I have frequently given large doses of quinine alone for the arrest of uterine hemorrhage and have never yet seen any appreciable decrease of the flow from its use. Neither do I believe it has any effect on the pregnant uterus unless given in doses sufficiently large to severely irritate the kidneys and bladder.

It is true that we sometimes see women abort after taking quinine for the cure of malarial fever; but here we are in danger of mistaking a post hoc for a propter hoc. It is well known that chill and fever will, unless promptly arrested, frequently produce abortion. Within the last two weeks I have arrested a threatened abortion produced by malarial fever, by the prompt exhibition of quinine; and that too without combining it with morphine; and in those cases, if any such occur, in which there is no element of malarial disease, and abortion follows the exhibition of quinine, it seems as if it might be ascribed to the individual peculiarity. On one occasion I gave to a pregnant woman forty-seven grains of quinine for the cure of neuralgia, without its producing any ill effects. Heat to the spine seems to produce contraction of the smaller vessels of the lower extremities and pelvic viscera, causing a sensation of cold of which patients frequently complain. I am indebted to Dr. Chas Duffey, Jr., for the idea of applying heat to the spine for the arrest of uterine hemorrhage.

Fourth. A condom tied over the female nozzle of a Davidson syringe can be used for dilating the female urethra. The nozzle covered by the condom, is passed into the urethra, until one-half is in the bladder, while the other is outside. It is gradually pumped full of water until the urethra is stretched to the desired extent.

Fifth. As a vaginal tampon, the end of the condom is first carried up to the fornix vaginæ and then packed full of old rags, sponge, or anything convenient. Or it can be stretched over a cylindrical speculum and packed through the speculum, after the method of Scanzoni. The advantages of a condom as a tampon are manifold. It is cleanly, being water proof, none of its contents get soiled. It will admit of any amount of packing without causing the woman pain. It can be easily withdrawn by first pulling out its contents.

Sixth. As a means of applying ice to the vagina, the condom is introduced as for tamponing, and then filled with ice and the end securely tied, and no dribbling takes place. It can be easily removed after the ice melts, emptied and is again ready for use.

Seventh. As a means of applying ice to the cavity of the uterus for the arrest of post partum hemorrhage—the condom is first filled with ice, tied well, oiled, and passed into the cavity of the uterus. As many may be used as necessary.

Eighth. As a means of arresting post partum hemorrhage. For this purpose the condom, or, what is better, as it admits of very great distension, an india rubber balloon (which can be bought at any toy shop,) is tied over the end of a Davidson syringe nozzle, and passed into the cavity of the flaccid uterus. It is then distended with warm or cold water. By this means we bring pressure directly to bear upon the mouths of the bleeding vessels, which effectually closes them. It might be objected to this method, that after the uterus is distended it might refuse to contract; but this seems hardly probable; and even should such an event occur, after the woman has rallied, we can administer ergot in sufficient doses to produce contraction. As soon as the distension required to check the hemorrhage is reached, we then pull the tube from the syringe bulb, and compress it firmly between the thumb and forefinger. By so doing we have perfect control of the water in the balloon, and can allow it to escape *pari-passu* with the uterine contractions.

Ninth. As a uterine dilator, three or four condoms are passed one into the other, and then fastened over the female nozzle of a Davidson syringe. Thus prepared, the nozzle is passed through the os, until one-half is in the cavity of the uterus; it is then held in position while water is pumped into the condoms until the requisite amount of distension is reached. It has the advantage over other uterine dilators that it is easier to introduce, while its distensile power is equally great.

Tenth. As a means of applying ice to the rectum.

Eleventh. As a means of arresting hemorrhage from the rectum, the condom and syringe are used just as the syringe and balloon are for the arrest of uterine hemorrhage.

Twelfth. As a rectal stricture dilator. Whenever we wish to improvise a dilator, either for the gradual dilatation or forcible rupture of rectal stricture, we pass half a dozen condoms one into the other, and fasten them over the female nozzle, pulling them lengthwise, so as to cause them to lay close to the nozzle. It is best in such cases to perforate the syringe nozzle midway between the point and the screw end. If this be neglected, difficulty will sometimes be found to the entrance of water into the condom, on account of closure of the end of the nozzle by the tense rubber drawn over its point. The dilator thus prepared is passed half way through the stricture, and gradually filled with water, until the stricture is sufficiently dilated or forcibly ruptured, If the condoms, as soon as they begin to fill, should slip through the stricture, up the bowel, as they often will do if the stricture is within an inch or two of the anus, we can continue to fill them, and as soon as they attain the size of a large man's wrist, we can pull them through the stricture, and dilate it by this means equally as well as if they had remained in situ. The sphincter ani can be over distended and paralyzed by the same method.

Thirteenth. As a means of constant pressure for the cure of orchitis, the condom is slipped over the swollen testicle, and allowed to remain until the cure is effected. For this suggestion I am indebted to Dr. Black, of Fort Wayne, Ind. (See *American Medical Weekly*, Sept. 5th, 1874.) His method of applying it is by folding the condom back upon itself, after the manner of the inverted finger of a glove, and turning it over the inflamed testicle just as a tight sock is turned on the foot. But by whatever method you try to put it on you will find it difficult.

Fourteenth. As a syringe the condom is first filled with water and a reed tied into the mouth of it. We thus have a syringe after the old style reed, and pig's bladder.

Fifteenth. The syringe and condoms, prepared as for dilating rectal stricture, can be used for dilating the wound made in median or lateral lithotomy, or instead of Dolbeau's dilator, in cases of perineal lithotrity.

